# Draft Genome Sequences for Five *Photorhabdus* Bacterial Symbionts of Entomopathogenic *Heterorhabditis* Nematodes Isolated from India

**DOI:** 10.1128/MRA.01404-18

**Published:** 2019-01-24

**Authors:** Vishal Singh Somvanshi, Bhumika Dubay, Jyoti Kushwah, Sivakumar Ramamoorthy, Udayakumar S. Vishnu, Jagadesan Sankarasubramanian, Jeyaprakash Rajendhran, Uma Rao

**Affiliations:** a Division of Nematology, ICAR-Indian Agricultural Research Institute, New Delhi, India; b Department of Genetics, School of Biological Sciences, Madurai Kamaraj University, Madurai, Tamil Nadu, India; University of Delaware

## Abstract

Photorhabdus bacteria exhibit contrasting lifestyles; they are virulent insect pathogens but symbionts of the entomopathogenic Heterorhabditis nematodes. Photorhabdus genomes encode several secondary metabolites and insecticidal protein toxins. Here, we present the draft genome sequences for five Photorhabdus strains isolated from Heterorhabditis nematodes collected from various geographical regions of India.

## Abstract (precis)

Photorhabdus bacteria exhibit contrasting lifestyles; they are virulent insect pathogens but symbionts of the entomopathogenic Heterorhabditis nematodes. Photorhabdus genomes encode several secondary metabolites and insecticidal protein toxins.

## ANNOUNCEMENT

Photorhabdus spp. are Gram-negative gammaproteobacteria found in nature in association with the entomopathogenic nematodes of the genus Heterorhabditis (1, 2). The first Photorhabdus genome was sequenced in 2003 (3), and at present, 33 genome sequences of various Photorhabdus spp. are available in NCBI GenBank (https://www.ncbi.nlm.nih.gov/genome?term=Photorhabdus). The genus Photorhabdus was revised to include 15 species on the basis of whole-genome, biochemical, chemotaxonomic, and ribosomal protein fingerprinting information, i.e., P. bodei, P. australis, P. akhurstii, P. caribbeanensis, P. hainanensis, P. kayaii, P. kleinii, P. namnaonensis, P. noenieputensis, P. laumondii, P. cinerea, P. khanii, P. stackebrandtii, P. tasmaniensis, and P. thracensis (4). We previously isolated the symbiont bacteria from the infective juveniles (IJs) of the entomopathogenic nematodes isolated from various geographical locations in India (5, 6). Preliminary biochemical and virulence characterization suggested genetic variations between different isolates (6). The 16S rRNA gene marker identified them to be a member of erstwhile Photorhabdus luminescens species (Table 1). To ascertain the identity of these isolates and to investigate the reasons for the differences in biochemical characters and virulence (6), we sequenced the genomes of these isolates.

**TABLE 1:** Information and genome statistics for the sequenced *Photorhabdus* bacterial strains and comparison to the already sequenced and annotated reference genome of *Photorhabdus luminescens* subsp. *laumondii* TTO1^*a*^

Feature^*b*^	Data for strain:				
	*P. akhurstii* IARI-SGMG3	*P. akhurstii* IARI-SGHR2	*P. akhurstii* IARI-SGHR4	*P. akhurstii* IARI-SGMS1	*P. laumondii* subsp. *clarkei* IARI-SGHP1
Place of origin	Meghalaya (northeastern Himalayan region), India	Haryana (Trans-Gangetic Plains), India	Haryana (Trans-Gangetic Plains), India	Maharashtra (western plateau and hill region), India	Himachal Pradesh (northern Himalayan region), India
Nematode host	*Heterorhabditis* sp.	*Heterorhabditis indica*	*Heterorhabditis indica*	*Heterorhabditis indica*	*Heterorhabditis* sp.
16s rRNA gene accession no.	JX221722	KJ995730	JX221723	JX240394	
No. of reads	2,583,080	1,391,368	1,415,756	1,195,633	2,384,036
Total data generated (Mb)	540	288	297	255	486
Insert size (bp)	72	72	72	72	72
Genome size (bp)	5,663,704	5,514,710	5,414,651	5,395,311	5,403,536
Coverage (×)	96	51	53	45	86
No. of contigs	228	220	212	342	190
GC content (%)	42.9	42.7	42.5	42.6	42.5
No. of CDS	5,036	5,055	4,955	4,942	5,623
No. of RNAs	128	78	75	73	100
*N*_50_ (bp)	92,101	82,937	95,541	43,831	103,009
Predicted no. of genes	5,016	5,083	4,953	5,040	5,933
No. (%) of annotated genes	4,068 (81.1)	4,207 (82.7)	4,083 (82.4)	4,101 (81.4)	4,669 (78.6)
No. (%) of genes matched to reference genome	4,188 (83.5)	4,246 (83.5)	4,170 (84.2)	4,246 (84.2)	5,089 (85.8)
No. of genes annotated but absent in reference genome	332	333	386	368	218
No. of genes present in reference genome but not annotated	516	536	499	541	218
SRA accession no.	SRX3720927	SRX3720926	SRX3720929	SRX3720928	SRX3720925
WGS GenBank accession no.	PUWT00000000	PUWU00000000	PUWV00000000	PUWW00000000	PUWX00000000

a For all strains, Semiconductor sequencing using the Ion Torrent Personal Genome Machine was used, with 200-bp chemistry for library preparation, and MIRA version 4.0.2 with *de novo* assembly.

b CDS, coding sequences; WGS, whole-genome shotgun.

A single colony of each strain was inoculated in 5 ml of Luria-Bertani (LB) broth and grown at 28°C with agitation (200 rpm) for 12 h. The genomic DNA was isolated by using a DNeasy kit (Qiagen, Hilden, Germany). For sequencing library preparation, 100 ng of genomic DNA was sheared enzymatically for 3 to 4 min using an Ion Shear Plus kit. The sheared DNA was purified using AMPure beads (Beckman Coulter Life Sciences, Indianapolis, IN, USA) and ligated with barcoded adapters. Subsequently, the adaptor-ligated fragments were resolved on a 2% E-Gel (Thermo Fisher Scientific, Waltham, MA, USA), and ∼330-bp fragments were collected. The size-selected fragments were PCR amplified using adaptor-specific primers for five cycles using high-fidelity Platinum supermix provided in the Ion Plus fragment library kit (Thermo Fisher Scientific, Waltham, MA, USA). The amplified product was purified using AMPure beads, and this final library was used for template generation for sequencing. The whole-genome sequencing was performed by the Semiconductor sequencing using the Ion Torrent Personal Genome Machine (PGM) system.

The good-quality reads were exported using the FileExporter plugin in the Ion Torrent Personal Genome Machine-associated Torrent Suite software, using the default parameters. The *de novo* genome assembly, scaffold construction, and gap closure were done by using MIRA version 4.0.2 (7), with the default parameters and providing the technology as “Iontor.” The fold coverage was estimated using the total number of sequence reads divided by the estimated genome size. Gene prediction was made *ab initio* using GeneMarkS (8) with default parameters. These predicted genes were further mapped to the reference genome (P. luminescens subsp. laumondii TTO1, NCBI RefSeq accession number NC_005126) using Blast2GO (9), with an E value cutoff of 1.0E–3. The amino acid sequences of the predicted genes were matched with the nonredundant protein database and annotated with InterProScan using Blast2GO.

The sequencing generated 1.2 to 2.5 million reads, generating 255 to 540 Mb of total sequence data (Table 1). The *de novo* assembly resulted in final genome sizes of 5.3 to 5.6 Mb, with a coverage of 45 to 96× (Table 1). A total of 190 to 228 contigs were obtained, with an *N*_50_ value of 43 to 103 kb. The GC content of the Photorhabdus genomes was 42.5 to 42.7%. We predicted 4,953 to 5,933 genes in the sequenced Photorhabdus strains, of which 78.6 to 82.7% could be annotated. These genes showed 83.5 to 85.8% match to the reference P. luminescens subsp. laumondii TTO1 genome (Table 1).

### Data availability.

The data generated in this study can be accessed at NCBI under SRA study accession number SRP133050, BioProject number PRJNA434554, and SRA experiment accession numbers SRX3720925 to SRX3720929. The draft genome accession numbers are provided in Table 1.
